# Regulatory effects of lavender varieties on rhizosphere soil properties and fungal community structure and function in semi-arid regions

**DOI:** 10.3389/fmicb.2026.1869680

**Published:** 2026-09-04

**Authors:** Yongshang Tong, Yuefang Chen, Yule Li, Peiqi Duan, Shunjie Yang, Yujie Ma, Wenyan Ning, Pansheng Ge, Qianhui Li, Ruiying Qin, Na Li

**Affiliations:** 1 Xinjiang Key Laboratory of Lavender Conservation and Utilization, Yining, China; 2 College of Biological Science and Technology, Yili Normal University, Yining, China

**Keywords:** environmental driving factors, fungal community structure, lavender cultivars, rhizosphere soil microecology, semi-arid region

## Abstract

**Introduction:**

Understanding how different lavender cultivars regulate soil microecology in semi-arid regions will reveal microbial mechanisms of soil improvement, identify cultivars with high ecological restoration potential, and support coordinated vegetation restoration, soil quality enhancement, and specialty crop cultivation.

**Methods:**

This study selected three major cultivated varieties in the Ili region of Xinjiang (Xinxun No. 2, Xinxun No. 3, and Xinxun No. 4) along with natural soil as a control. The physicochemical properties of rhizosphere soil were determined, and high-throughput sequencing technology was used to analyze the fungal community structure, while fungal functions were predicted using FUNGuild. Correlation analysis and redundancy analysis (RDA) were further applied to identify key driving factors.

**Results:**

Different varieties significantly reshaped rhizosphere soil properties and fungal community structure. Among them, Xinxun No. 4 exhibited the most pronounced increases in soil organic matter, available nitrogen, and available phosphorus, and significantly enhanced fungal alpha diversity. The fungal community was dominated by Ascomycota; however, species abundance at the phylum, genus, and putative functional group levels differed significantly among varieties. Redundancy analysis indicated that soil total phosphorus, pH, and total nitrogen were the key factors influencing fungal community structure, whereas alkali-hydrolyzable nitrogen, available phosphorus, organic matter, electrical conductivity, total phosphorus, and nitrate nitrogen were the main driving factors affecting putative fungal functional groups.

**Discussion:**

Xinxun No. 4 was relatively advantageous in improving soil nutrient status and fungal community structure, and thus may be preferentially considered for promotion in applications such as lavender garden establishment.

## 1 Introduction

Lavandula spp. is an important aromatic plant that combines economic value with ecological functions, and it is widely used in essential oil extraction, pharmaceuticals and health care, cosmetics, and food processing (Cheng et al., 2024; Dong et al., 2024). In recent years, with the rapid development of the aromatic industry and ecological agriculture, lavender has become one of the characteristic and advantageous economic crops in arid and semi-arid regions (Yetik and Sen, 2025). Benefiting from abundant sunlight and heat resources, a relatively large diurnal temperature range, and suitable arid to semi-arid climatic conditions, the Yili region of Xinjiang, China, has developed into a major core production area for large-scale lavender cultivation in China (Li et al., 2024). However, under long-term intensive cultivation, different lavender cultivars show pronounced differences in yield, quality, and environmental adaptability (Lin et al., 2024; Lin et al., 2025; Zeng et al., 2025). Such variation is influenced not only by genetic background, but also closely associated with the structure and function of their rhizosphere microecological systems (Wen et al., 2025). Therefore, elucidating the mechanisms underlying inter-cultivar variation in lavender from the perspective of rhizosphere ecological processes is of great significance for improving cultivation efficiency and ecological adaptability.

The rhizosphere is the most active zone of interaction between plant roots and the soil environment, and it serves as a key interface through which plants regulate soil microbial communities and maintain nutrient cycling and soil health (Solomon et al., 2024; Pang et al., 2026). Plants can alter the chemical environment of the rhizosphere through root exudates, thereby selectively recruiting specific microbial taxa and forming cultivar-specific rhizosphere microbiomes (Tsai et al., 2025; Yang et al., 2025). In recent years, advances in high-throughput sequencing technologies have deepened research on soil fungal communities. As an important component of soil ecosystems, fungi not only participate in the decomposition of organic matter and the cycling of nutrients such as carbon, nitrogen, and phosphorus, but also influence plant growth and health through symbiotic, parasitic, or antagonistic interactions (Han et al., 2025; Martin and Tan, 2025). Among them, arbuscular mycorrhizal fungi can enhance plant acquisition of nutrients and water, whereas certain pathogenic fungi may cause root diseases and reduce crop yield (Ahmed et al., 2026). Previous studies have shown that different plant species, and even different cultivars within the same species, can significantly influence the assembly process and functional differentiation of rhizosphere microbial communities through changes in the composition and quantity of root exudates (Hou et al., 2026). However, research on how different lavender cultivars shape the structure and ecological functions of rhizosphere fungal communities remains limited, and the underlying mechanisms are still not systematically understood.

Soil physicochemical properties serve as an important bridge linking plant root activity with microbial responses (Philippot et al., 2024; Zhang X. Y. et al., 2026). Factors such as soil pH, organic matter, total nitrogen, available phosphorus, and soil moisture not only directly affect fungal growth, reproduction, and community composition, but are also feedback-regulated by plant root uptake, exudation, and nutrient redistribution processes (Yao et al., 2025; Wu et al., 2026). Different lavender cultivars vary in nutrient-use efficiency, root growth traits, and the secretion of secondary metabolites; these differences may further drive the restructuring of fungal community composition and the shifts of functional groups by altering rhizosphere soil nutrient status and microenvironmental conditions (Ping et al., 2024; Peng et al., 2025). This effect may be especially pronounced in arid and semi-arid regions, where limited water availability and low organic matter background can amplify the selective pressure of plants on rhizosphere microorganisms, thereby influencing the ecological functions of fungal communities (Qi et al., 2026). However, research on the coupling relationship among “plant cultivar–soil physicochemical properties–fungal community structure and function” remains insufficient, particularly in typical dryland cash-crop systems, where systematic evidence is still lacking.

Existing lavender studies have focused mainly on agronomic traits, essential oil yield, and quality differences(Cheng et al., 2024; Dong et al., 2024; Yetik and Sen, 2025), whereas comparatively little attention has been paid to the regulatory effects of different cultivars on the soil microenvironment and microbial communities from a rhizosphere ecology perspective. Given that the native soil background itself exerts an important influence on microbial communities, comparing rhizosphere soils after lavender cultivation with uncultivated natural soils can help distinguish plant-driven effects from environmental background effects, thereby more accurately revealing cultivar-specific rhizosphere regulatory mechanisms. Therefore, systematically evaluating the effects of different lavender cultivars on rhizosphere soil properties, fungal community structure, and function under a unified ecological background will not only deepen our understanding of rhizosphere ecological processes in aromatic plants, but also provide a theoretical basis for superior cultivar screening and optimized soil management.

Based on this, this study takes three major lavender cultivars grown in the Ili region of Xinjiang—Xinxun No. 2, Xinxun No. 3, and Xinxun No. 4—as well as natural soil as the research objects, and addresses the following scientific questions: (1) Do different lavender cultivars significantly affect rhizosphere soil physicochemical properties and fungal community structure? (2) Which soil environmental factors are the key drivers of changes in fungal community structure and putative functional groups? (3) What are the relationships between lavender rhizosphere soil properties and fungal community structure as well as predicted functions? This study aims to reveal the ecological mechanisms by which cultivar differences drive rhizosphere microbiome assembly in lavender, and to provide a scientific basis for lavender cultivar selection, soil health maintenance, and sustainable cultivation management in arid and semi-arid regions.

## 2 Materials and methods

### 2.1 Study area overview

The soils used in this study were collected from the lavender planting base of the Institute of Agricultural Sciences, the Fourth Division of the Xinjiang Production and Construction Corps (44°03′N, 81°32′E), at an altitude of approximately 650 m. The area has a temperate continental semi-arid climate, abundant sunshine, annual sunshine duration of up to 3,500 h, a frost-free period of more than 150 days, and an average annual precipitation of 250 mm. The soil type is gray-calcic soil, characterized by loose texture and relatively flat terrain, with consistent irrigation and field management conditions. The lavender plants used in this study were grown at the same base and were all 5-year-old established plants, with essentially the same cultivation management practices, fertilization regime, and irrigation conditions. Natural soil from the same plot, where lavender had not been planted, was used as the control (CK) to compare the effects of different lavender cultivars on the rhizosphere fungal community.

### 2.2 Experimental design

In this study, three lavender cultivars were selected as the research objects, namely Xinxun No. 2, Xinxun No. 3, and Xinxun No. 4. Four treatments were established: Xinxun No. 2 (XL2), Xinxun No. 3 (XL3), Xinxun No. 4 (XL4), and a natural soil control (CK). The lavender planting area was arranged in a randomized block design. For each lavender cultivar., five independent planting blocks were established, with each block measuring 30 m × 50 m. The distance between different blocks was 15 m, with road barriers placed between them, while the distance between adjacent plots within the same block was 5 m. The natural soil control group was established in an area more than 15 m away from the lavender planting area.

### 2.3 Collection and processing of rhizosphere soil samples

Sampling was conducted at the full flowering stage, and all treatment samples were collected on the same day to avoid potential effects of differences in sampling time on soil physicochemical properties and microbial communities. After excluding potential edge effects, five healthy plants with similar growth vigor and no obvious signs of pests or diseases were randomly selected from each independent replicate plot as subsamples within the plot. Before sampling, fallen leaves and weeds around the base of each plant were removed. During sampling, the complete root system was excavated from around the plant base. Loosely attached non-rhizosphere soil was first gently shaken off from the roots, and the soil still tightly adhering to the root surface was subsequently defined as rhizosphere soil. Rhizosphere soil was separated from the root surface by gently brushing with a sterile brush and then collected. The rhizosphere soil obtained from the five plants within each replicate plot was thoroughly mixed to form one plot-level composite sample, representing one biological replicate. Approximately 200 g of soil was collected for each composite replicate sample. During sampling, the sampling tools were replaced or cleaned between different sampling points, replicate plots, and treatments, and were disinfected by wiping with 75% ethanol to minimize cross-contamination. All samples were immediately placed in sterile self-sealing bags after collection, stored at low temperature, and transported back to the laboratory as soon as possible. Control soil samples (CK) were collected from natural soil without lavender cultivation within the same experimental area, with sampling sites located more than 15 m away from the lavender planting area. For the CK samples, soil from the 0–20 cm layer, corresponding to the main distribution range of lavender roots, was collected using the five-point sampling method within five pre-established 10 m × 10 m quadrats. Soil collected from each CK replicate plot was similarly mixed to form an independent composite sample. After being transported to the laboratory, all soil samples were passed through a 2 mm sieve and divided into subsamples. One portion was naturally air-dried for the determination of soil physicochemical properties, while the other portion was stored at −80 °C for subsequent DNA extraction and high-throughput sequencing analysis.

### 2.4 Determination of soil physicochemical properties

Soil pH was determined using the glass electrode method (soil-to-water ratio, 1:2.5). Electrical conductivity (EC) was measured with a conductivity meter (soil-to-water ratio, 1:5). The contents of ammonium nitrogen (NH_4_^+^-N) and nitrate nitrogen (NO_3_^−^-N) were determined using an automatic discrete chemical analyzer (Angot et al., 2025). Soil organic matter (SOM) was measured by the potassium dichromate oxidation–external heating method (Komy, 1995). Total phosphorus (TP) was determined by the alkaline digestion–molybdenum antimony colorimetric method (Parkinson and Allen, 2009). Available nitrogen (AN) was measured based on the alkaline hydrolysis–diffusion method (Zhou et al., 2024). Available phosphorus (AP) was determined by the sodium bicarbonate extraction-spectrophotometric method (Chen et al., 2025). Total nitrogen (TN) was measured using an elemental analyzer (Wild et al., 2024).

### 2.5 Soil DNA extraction, PCR amplification, and high-throughput sequencing

A 0.5 g aliquot of rhizosphere soil stored at −80 °C was weighed, and total soil DNA was extracted using the E. Z. N. A.® Soil DNA Kit (Omega Bio-tek, Norcross, GA, USA). The extracted DNA was assessed for integrity by 1% agarose gel electrophoresis, and its concentration and purity were measured using a spectrophotometer or fluorometer. PCR amplification was performed using the fungal ITS rDNA forward primer ITS1F (5′-CTTGGTCATTTAGAGGAA GTAA-3′) and reverse primer ITS2R (5′-GCTGCGTTCTTCATCGA TGC-3′) (Han et al., 2025). After purification and quantification, the PCR products were pooled in equimolar amounts to construct sequencing libraries, followed by paired-end sequencing on the Illumina NextSeq 2000 (Illumina, United States). The raw sequencing reads were deposited into the GSA Sequence Read Archive (SRA) database (Accession Number: CRA041854).

### 2.6 Sequencing data processing and fungal community analysis

Raw paired-end sequencing reads were quality-controlled using fastp (https://github.com/OpenGene/fastp, version 0.23.4) and merged using FLASH (http://www.cbcb.umd.edu/software/flash, version 1.2.11). The main procedures were as follows: (1) bases with a quality score below 20 at the 3′ end of reads were filtered using a 50-bp sliding window. If the average quality score within the window was lower than 20, the read was truncated from the beginning of that window. Reads shorter than 50 bp after quality control were discarded, and reads containing more than five N bases were removed; (2) paired-end reads were merged into single sequences according to their overlap, with a minimum overlap length of 10 bp; (3) the maximum mismatch ratio allowed in the overlap region of the merged sequences was 0.2, and sequences that did not meet this criterion were discarded; and (4) samples were distinguished according to the barcodes and primers at both ends of the sequences, and sequence orientations were adjusted. No mismatches were allowed in the barcodes, and a maximum of two mismatches were allowed in the primers. The quality-filtered and merged sequences were clustered into operational taxonomic units (OTUs) at 97% sequence identity using USEARCH (http://drive5.com/usearch/, version 11), and chimeric sequences were removed. Taxonomic annotation of OTUs was performed using RDP Classifier (http://rdp.cme.msu.edu/, version 2.11) against the UNITE 9.0/ITS_fungi database, with a confidence threshold of 70%. To minimize the influence of sequencing depth on subsequent analyses, the abundance data of the 20 samples were rarefied to an equal sequencing depth of 68,144 sequences per sample, resulting in a total of 5,765 OTUs. Based on these data, alpha diversity indices, including Chao1, Shannon, Simpson, and Ace, as well as beta diversity metrics, were calculated. Principal coordinate analysis (PCoA) and related methods were then used to analyze differences in rhizosphere fungal community structure among different lavender cultivars. In this study, 97% sequence similarity-based OTU clustering was used instead of the ASV approach, mainly for the following reasons. Although the ASV approach provides higher single-nucleotide resolution and better reproducibility across studies, it may split sequences belonging to the same species or to ecologically and functionally similar taxa into multiple sequence variants, thereby increasing uncertainty in the interpretation of community diversity and functional patterns. Therefore, the use of UPARSE for 97% OTU clustering is more consistent with the analytical objectives of this study, which focus on ecological differences among cultivars and functional responses of microbial communities.

### 2.7 Statistical analysis

The original data were organized using Excel 2019, and SPSS 26.0 was used to perform statistical analyses of soil physicochemical properties, fungal community diversity indices, and major putative fungal functional groups. Differences among treatments were compared using one-way analysis of variance (one-way ANOVA). Putative fungal functional groups of soil fungi were annotated and predicted using FUNGuild, and their ecological responses were analyzed in conjunction with soil physicochemical properties. For FUNGuild-based functional annotation, the confidence ranking of each guild assignment was recorded. FUNGuild classifies assignments into three confidence levels: “Possible,” “Probable,” and “Highly Probable.” To reduce uncertainty and avoid overinterpretation of low-confidence ecological assignments, only OTUs assigned at the “Probable” or “Highly Probable” confidence levels were retained for subsequent quantitative analyses of trophic modes and functional guilds. Assignments classified as “Possible” were treated as low-confidence predictions and were not included in the main statistical analyses, but were considered only as auxiliary information. OTUs without FUNGuild annotations or with ambiguous low-confidence assignments were treated as unassigned. Pearson correlation analysis was conducted to reveal the relationships among soil factors, fungal community structure, and putative fungal functional groups, and redundancy analysis (RDA) was used to identify the key environmental factors driving changes in fungal community structure and putative fungal function. All figures and tables were prepared using Origin Pro 2025.

## 3 Results

### 3.1 Comparison of soil physicochemical properties

**Figure 1:**
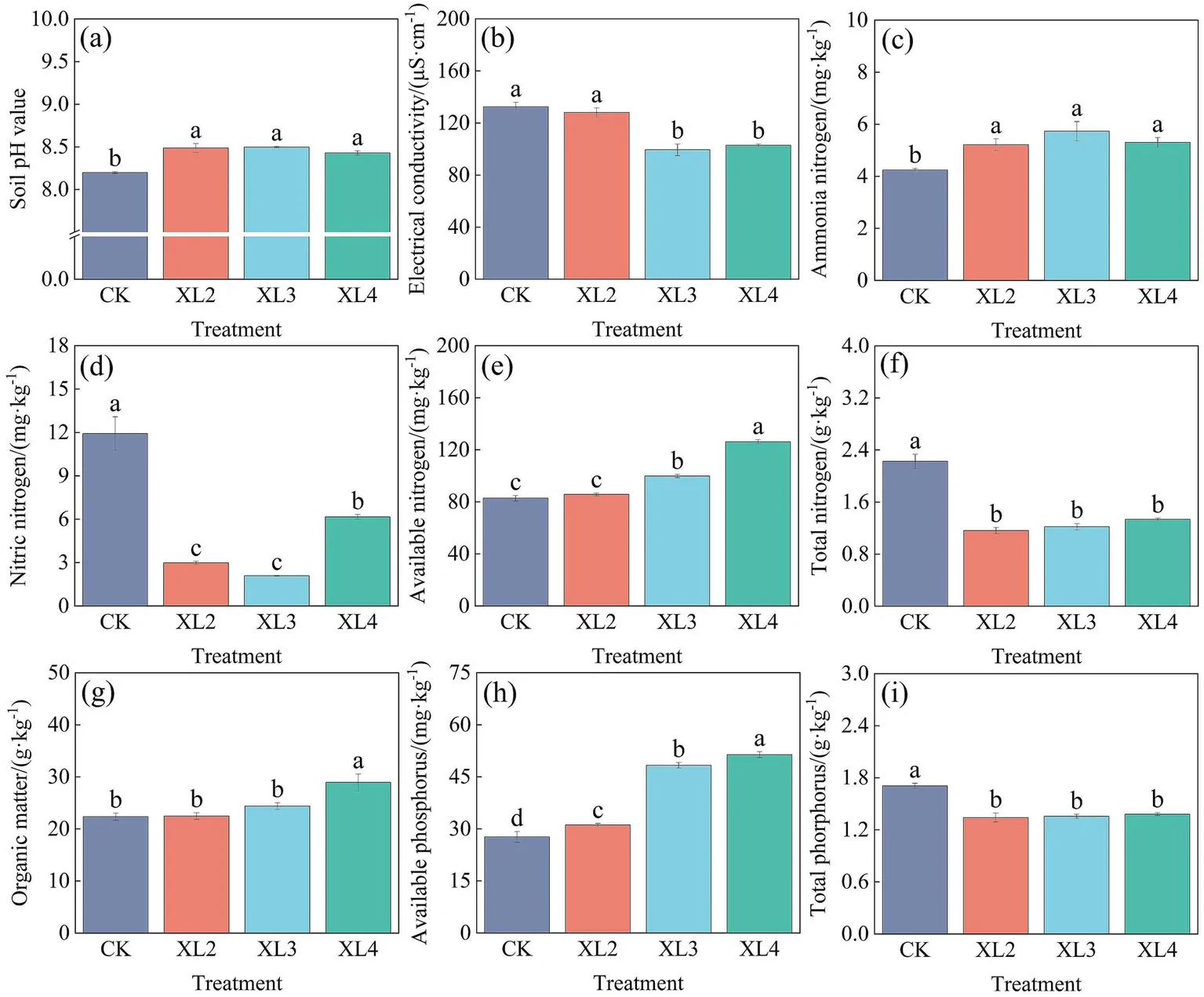
Comparison of soil physicochemical properties. Different lowercase letters indicate significant differences among treatments. **(a)** Soil pH value. **(b)** Electrical conductivity. **(c)** Ammonia nitrogen. **(d)** Nitric nitrogen. **(e)** Available nitrogen. **(f)** Total nitrogen. **(g)** Organic matter. **(h)** Available phosphorus. **(i)** Total phosphorus. CK represents the natural soil control group; XL2 represents the rhizosphere soil of Xinxun No. 2; XL3 represents the rhizosphere soil of Xinxun No. 3; and XL4 represents the rhizosphere soil of Xinxun No. 4.

Figure 1 shows that the physicochemical properties of the rhizosphere soil of different lavender varieties differed significantly from those of the natural soil control group (*p* < 0.05). Compared with the natural soil, the pH values of XL2, XL3, and XL4 increased by 3.54, 3.66, and 2.80%, respectively, all showing significant increases (Figure 1a). Electrical conductivity did not change significantly in XL2, whereas it decreased by 24.93 and 22.34% in XL3 and XL4, respectively, with significant differences compared with the natural soil and XL2 (Figure 1b). The ammonium nitrogen content in XL2, XL3, and XL4 increased by 23.11, 35.09, and 25.00%, respectively, all significantly higher than that of the natural soil (Figure 1c); in contrast, nitric nitrogen decreased markedly by 75.00, 82.45, and 48.32%, respectively (Figure 1d). Available nitrogen increased by 20.74 and 52.50% in Xinxun No. 3 and Xinxun No. 4, respectively, while Xinxun No. 2 did not differ significantly from the natural soil (Figure 1e). Compared with the natural soil control group, total nitrogen content in XL2, XL3, and XL4 decreased by 40.36–47.98% (Figure 1f). Among the treatments, Xinxun No. 4 had the highest organic matter content, increasing by 29.54% relative to the natural soil control group and significantly exceeding the other treatments (Figure 1g). The increase in available phosphorus was most pronounced: Xinxun No. 2, No. 3, and No. 4 increased by 12.38, 74.56, and 85.53%, respectively, with Xinxun No. 4 showing the highest value (Figure 1h). Compared with the natural soil control group, total phosphorus content in XL2, XL3, and XL4 decreased by 19.30–21.64% (Figure 1i). Overall, Xinxun No. 4 performed best in enhancing organic matter, available nitrogen, and available phosphorus, indicating a stronger promoting effect on soil nutrient accumulation and fertility improvement.

### 3.2 Differences in fungal community structure

**Figure 2:**
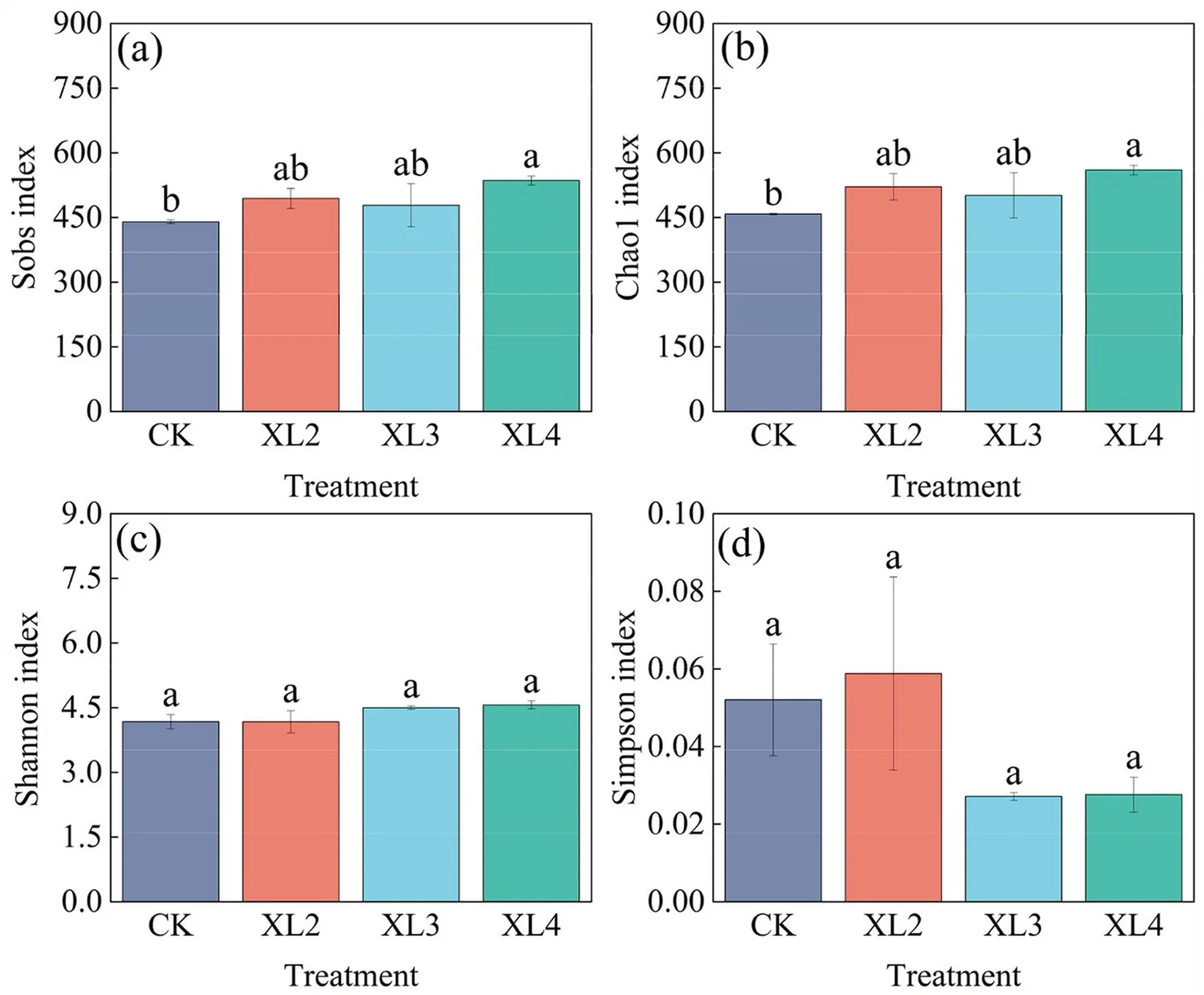
Comparison of soil fungal *α*-diversity. Different lowercase letters indicate significant differences among treatments. CK represents the natural soil control group; XL2 represents the rhizosphere soil of Xinxun No. 2; XL3 represents the rhizosphere soil of Xinxun No. 3; and XL4 represents the rhizosphere soil of Xinxun No. 4.

According to the analysis of fungal *α*-diversity data, the different habitat types showed differences in the Sobs, Shannon, Simpson, and Chao1 indices (Figure 2). The Xinxun No. 4 habitat exhibited relatively high fungal diversity, with the Sobs and Chao1 indices all being significantly higher than those of the natural soil control group and the other habitat types. For example, its mean Sobs value was 535.8, markedly higher than the natural soil control group’s 440 (Figure 2a). In addition, compared with the CK, Xinxun No. 2, and Xinxun No. 3 treatments, the Chao1 index of Xinxun No. 4 increased by 22.30, 7.50, and 11.75% (Figure 2b), respectively, while the Shannon index increased by 9.33, 9.36, and 1.49% (Figure 2c), respectively. This indicates that the fungal community in the Xinxun No. 4 habitat was richer and more diverse, which may be attributable to environmental conditions more favorable to fungal growth. The consistency across the diversity indices further strengthens the reliability of this conclusion.

**Figure 3:**
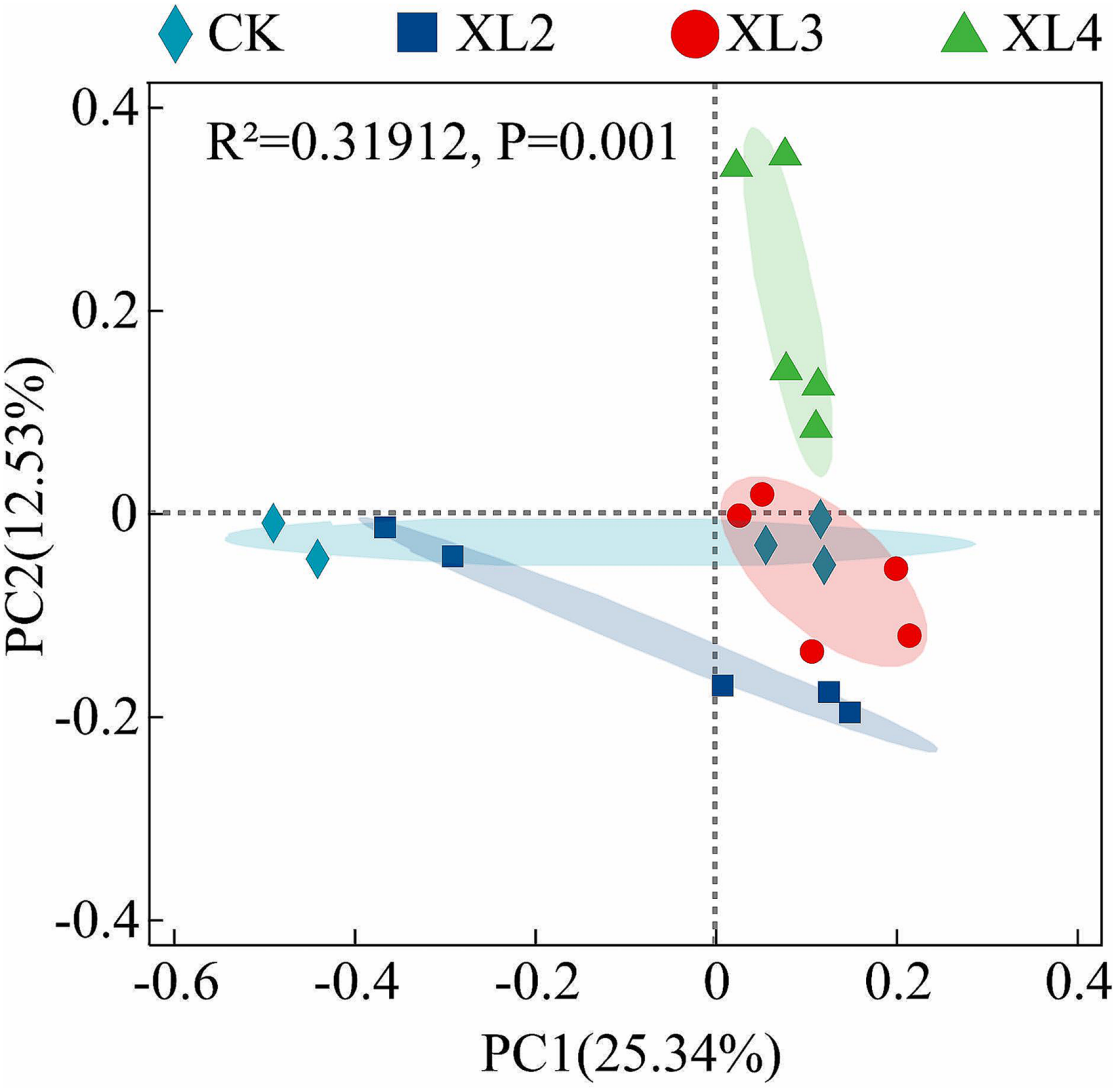
Principal coordinate analysis of soil fungal community composition at the OTU level. CK represents the natural soil control group; XL2 represents the rhizosphere soil of Xinxun No. 2; XL3 represents the rhizosphere soil of Xinxun No. 3; and XL4 represents the rhizosphere soil of Xinxun No. 4.

At the OTU level, the principal coordinate analysis (PCoA) results showed that the collected soil samples exhibited a clear separation trend in ordination space (Figure 3). The first two principal coordinate axes explained 25.34 and 12.53% of the community variation, respectively, with a cumulative explanatory rate of 37.87%, indicating that the two-dimensional ordination could, to a certain extent, reflect differences in soil fungal community structure among the different treatments. Overall, the CK and XL2 samples were mainly distributed in the negative region of PC1, while the XL3 samples were located more in the positive region of PC1. The XL4 samples, in contrast, showed a relatively independent clustering pattern along the positive direction of PC2, suggesting that the different treatments exerted a strong shaping effect on the composition of the soil fungal community.

**Figure 4:**
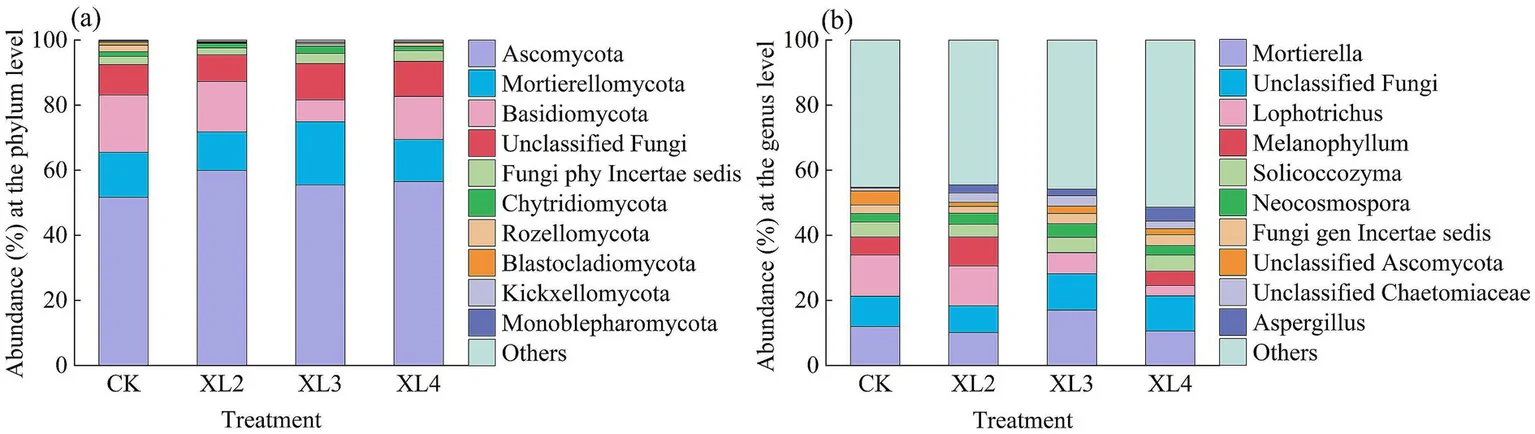
Comparison of the relative abundance of fungal community composition at the phylum level **(a)** and genus level **(b)**. CK represents the natural soil control group; XL2 represents the rhizosphere soil of Xinxun No. 2; XL3 represents the rhizosphere soil of Xinxun No. 3; and XL4 represents the rhizosphere soil of Xinxun No. 4.

As shown in Figure 4, the relative abundances of fungal taxa at both the phylum and genus levels exhibited marked differences among the different treatments. At the phylum level, all treatment samples were dominated by Ascomycota, which maintained a consistently high relative abundance across treatments (Figure 4a). Mortierellomycota and Basidiomycota were the next most abundant phyla, and their relative proportions varied somewhat among treatments. Overall, the proportion of Ascomycota was relatively higher in the XL2 and XL4 treatments, whereas Mortierellomycota increased and Basidiomycota decreased in the XL3 treatment, indicating that the treatment factor had a clear regulatory effect on the fungal community structure at the phylum level. At the genus level, Others accounted for a relatively large proportion, suggesting that many sequences in the samples had not yet been precisely annotated (Figure 4b). Among the annotated genera, Mortierella was the dominant genus shared by all treatments. In addition, Lophotrichus, Melanophyllum, Solicoccozyma, Neocosmospora, and several other genera collectively constituted the main dominant fungal groups. The relative abundances of these dominant fungal phyla differed significantly among the treatment groups. For example, compared with the CK treatment, the XL3 treatment significantly reduced the relative abundances of Basidiomycota, Rozellomycota, and Blastocladiomycota by 61.54, 64.21, and 97.25%, respectively. In contrast, the XL4 treatment significantly increased the relative abundance of Monoblepharomycota by 147.29%. These differences suggest that the various treatments exerted selective effects on the composition of the fungal community (see Table 1).

**Table 1:** Monte Carlo permutation test of the effects of soil factors on fungal community structure and putative fungal functions.

Indication	Community structure				Putative fungal functions			
	RDA1	RDA2	*r* ^2^	*p*	RDA1	RDA2	*r* ^2^	*p*
TP	−0.77	0.638	0.467	0.004	−0.857	−0.515	0.325	0.034
pH	0.681	−0.732	0.380	0.018	0.766	0.643	0.206	0.140
TN	−0.715	0.699	0.289	0.044	−0.802	−0.597	0.186	0.175
NO_3_^−^-N	−0.917	0.399	0.274	0.056	−0.548	−0.837	0.298	0.045
EC	−0.979	0.205	0.279	0.057	−0.988	0.157	0.406	0.012
AP	0.994	0.113	0.281	0.063	0.942	−0.335	0.544	0.002
NH_4_^+^-N	0.419	0.908	0.213	0.120	0.968	0.251	0.081	0.504
AN	0.999	−0.036	0.109	0.367	0.802	−0.598	0.801	0.001
SOM	0.992	0.129	0.068	0.530	0.811	−0.585	0.498	0.002

RDA1 indicates the cosine value of the angle between soil factor and ordination axis, and RDA2 indicates the correlation between soil factor and ordination axis. *r*^2^ is the determination coefficient of soil factors in species distribution. The smaller *r*^2^, the smaller the influence of soil factors on species distribution. *p* is the significance test of correlation.

**Figure 5:**
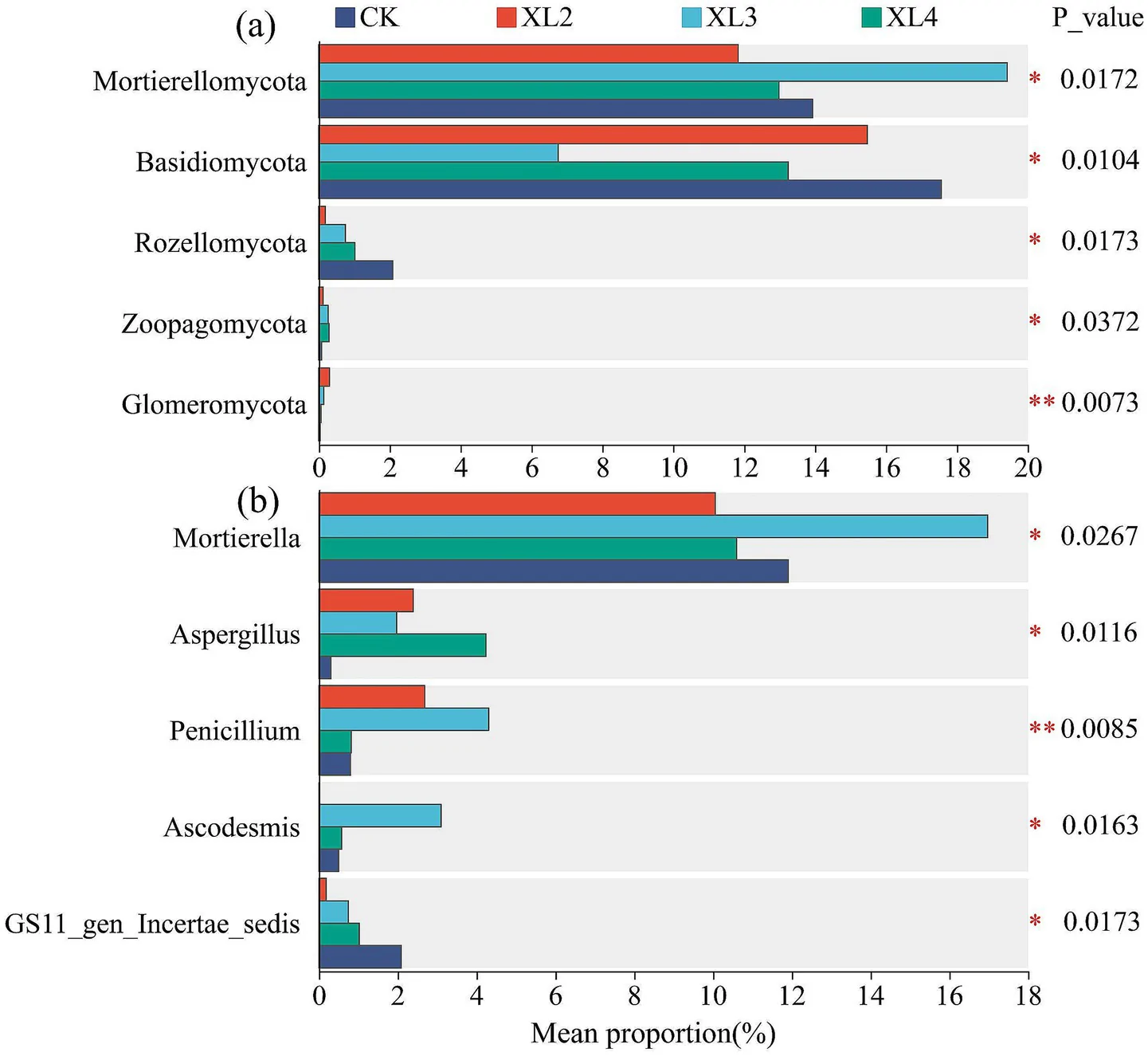
Comparison of dominant fungal phyla **(a)** and dominant fungal genera **(b)** showing significant differences among different treatments. CK represents the natural soil control group; XL2 represents the rhizosphere soil of Xinxun No. 2; XL3 represents the rhizosphere soil of Xinxun No. 3; and XL4 represents the rhizosphere soil of Xinxun No. 4.

Further analysis of Figure 5 shows that, at the phylum level, Mortierellomycota, Basidiomycota, Rozellomycota, Zoopagomycota, and Glomeromycota all exhibited significant differences among treatments (*p* < 0.05). Among them, Mortierellomycota showed the highest relative abundance in the XL3 treatment, whereas Basidiomycota was relatively more abundant in the CK group. This suggests that different lavender varieties may promote the enrichment of specific fungal taxa and reshape the community structure (Figure 5a). At the genus level, Mortierella maintained a relatively high abundance across all treatments and increased further in the XL3 treatment. Meanwhile, Aspergillus, Penicillium, Ascodesmis, and GS11_gen_Incertae_sedis showed significant fluctuations among treatments (*p* < 0.05), indicating that different lavender varieties exerted a strong selective effect on dominant fungal genera (Figure 5b).

**Figure 6:**
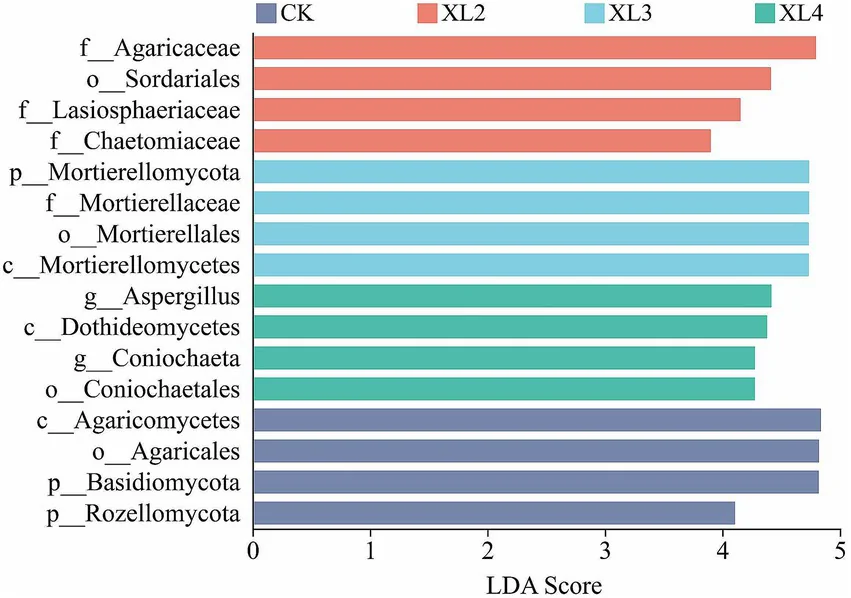
Soil fungal community LEfSe analysis: LDA score distribution bar chart (LDA = 3.5). CK represents the natural soil control group; XL2 represents the rhizosphere soil of Xinxun No. 2; XL3 represents the rhizosphere soil of Xinxun No. 3; and XL4 represents the rhizosphere soil of Xinxun No. 4.

LEfSe analysis showed that, using an LDA score threshold of 3.5, the soil fungal communities under different treatment groups exhibited significant differences across multiple taxonomic levels (Figure 6). Specifically, the differential biomarkers in the CK group were mainly concentrated in c_Agaricomycetes; the XL2 group was primarily enriched in f_Agaricaceae; the dominant differential taxon in the XL3 group was p_Mortierellomycota; and the XL4 group was mainly enriched in g_Aspergillus. These results indicate that different lavender varieties exerted a clear directional regulatory effect on the structure of the soil fungal community.

### 3.3 Fungal functional prediction

**Figure 7:**
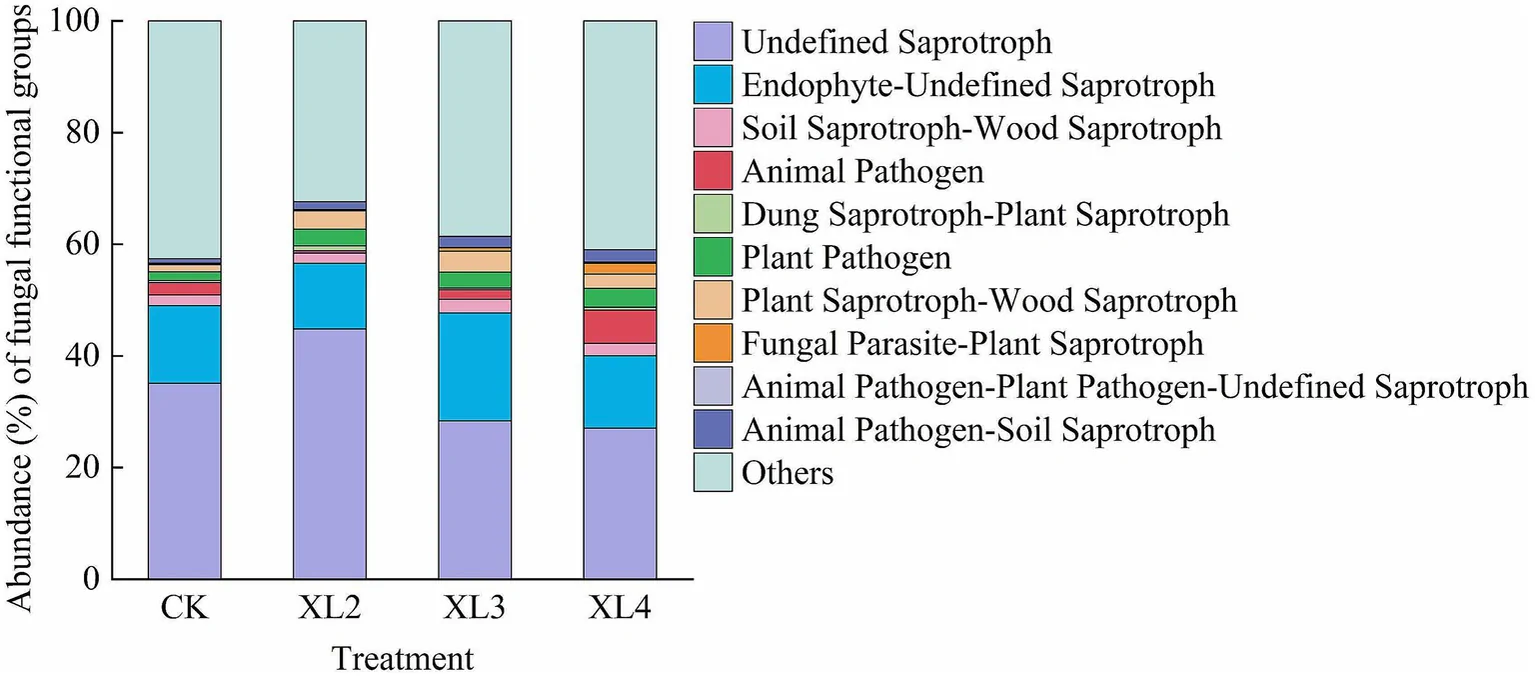
FUNGuild-based prediction of soil fungal community functional groups. Only guild assignments with “Probable” or “Highly Probable” confidence rankings were included in the quantitative comparison; “Possible” assignments were treated as low-confidence predictions and excluded from the main analysis. CK represents the natural soil control group; XL2 represents the rhizosphere soil of Xinxun No. 2; XL3 represents the rhizosphere soil of Xinxun No. 3; and XL4 represents the rhizosphere soil of Xinxun No. 4.

Based on the FUNGuild prediction analysis of soil fungal community functional guilds (Figure 7), the results showed that the composition of fungal trophic modes differed to some extent among treatments, but overall the communities were dominated by Undefined Saprotroph, Endophyte-Undefined Saprotroph, and Soil Saprotroph-Wood Saprotroph, which together accounted for a relatively high proportion of the total relative abundance. Compared with CK, the XL2 treatment significantly increased the relative abundance of Undefined Saprotroph to 44.79%, whereas the XL3 and XL4 treatments reduced the relative abundance of this putative fungal functional group to 28.35 and 27.05%, respectively. For Endophyte–Undefined Saprotroph, the XL3 treatment showed marked enrichment, with its relative abundance reaching 19.33%, representing a 38.9% increase compared with CK; by contrast, the XL2 and XL4 treatments showed decreasing trends, with relative abundances of 11.78 and 12.97%, respectively. In addition, the relative abundance of potential pathogenic or parasitic putative fungal functional groups, such as Animal Pathogen, Plant Pathogen, and Fungal Parasite-Plant Saprotroph, increased slightly in XL4. Overall, different treatments regulated the predicted functional composition of the soil fungal community: XL2 tended to promote the enrichment of saprotrophic functional guilds, whereas XL3 and XL4 showed a trend toward greater functional diversity and a higher proportion of pathogen-related guilds.

### 3.4 Correlation analysis between the fungal community and environmental factors

**Figure 8:**
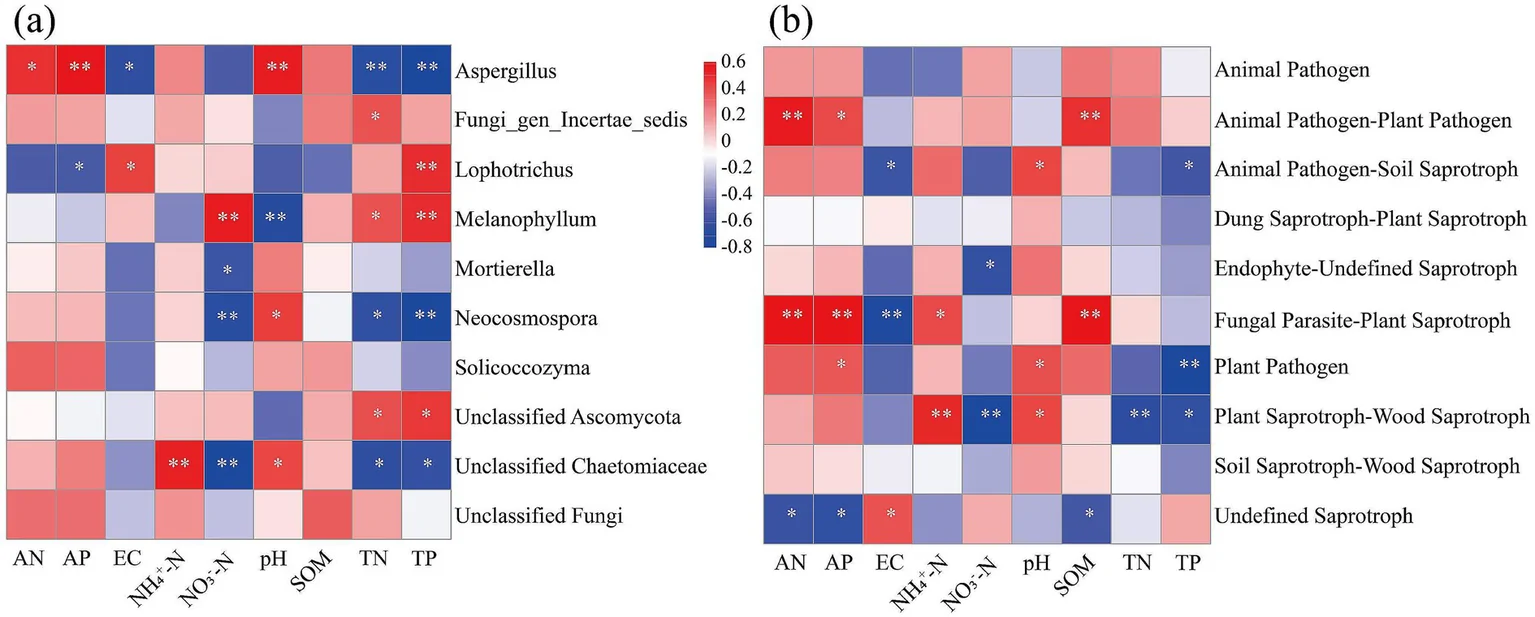
Correlation analysis between soil environmental factors and fungal community composition **(a)** and putative fungal functional groups **(b)**. AN, available nitrogen; AP, available phosphorus; EC, Electrical Conductivity; NH_4_^+^-N, soil ammonium nitrogen; NO_3_^−^-N, soil nitrate nitrogen; SOM, soil organic matter; TN, total nitrogen; TP: total phosphorus.

As shown in Figure 8, there are significant correlations between soil environmental factors and both the composition of fungal communities and the distribution of putative fungal functional groups. Different taxa exhibit distinct response patterns along environmental gradients. Overall, the composition of fungal communities at the genus level is closely associated with soil nutrient status, physicochemical properties, and forms of inorganic nitrogen. Some taxa show significant positive or negative correlations with AN, AP, EC, NH₄^+^-N, NO₃^−^-N, pH, SOM, TN, and TP (Figure 8a), indicating that soil nutrient supply capacity, acidity–alkalinity conditions, and salinity collectively contribute to the formation and differentiation of fungal community structure.

Further correlation analysis of putative fungal functional groups reveals that different ecological functional groups vary in both the direction and strength of their responses to soil environmental factors. Notably, pathogenic, saprotrophic, and multifunctional groups display pronounced differences in their correlations with these factors (Figure 8b), suggesting that soil physicochemical properties not only shape the taxonomic composition of fungal communities but may also drive the reorganization of their ecological functions. In summary, soil nutrient levels, pH, and inorganic nitrogen content are likely key environmental drivers influencing both the composition of fungal communities and the distribution of their functional groups.

**Figure 9:**
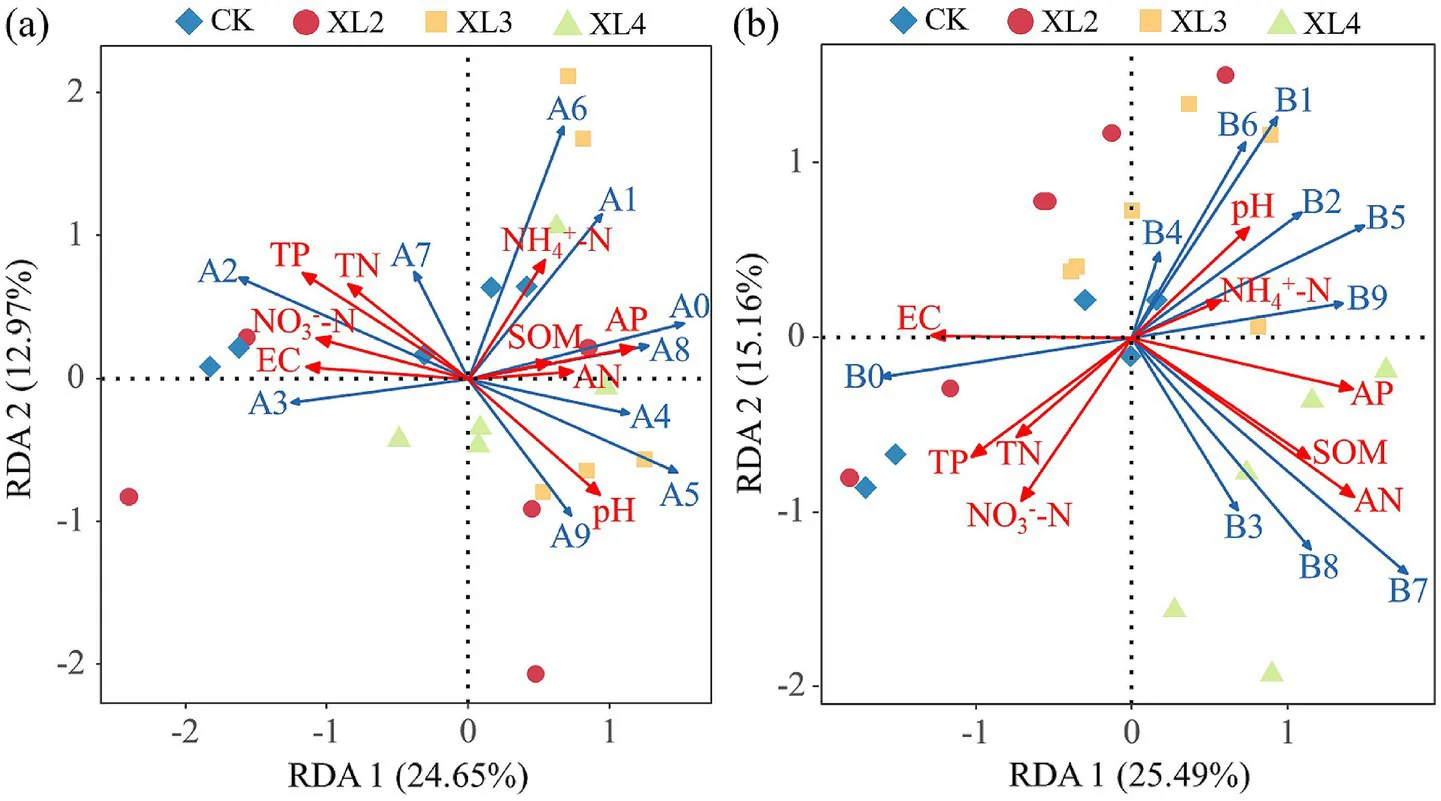
Redundancy analysis (RDA) of soil environmental factors in relation to fungal community composition **(a)** and putative fungal functional groups **(b)**. AN, Available nitrogen; AP, Available phosphorus; EC, Electrical conductivity; NH_4_^+^-N, Soil ammonium nitrogen; NO_3_^−^-N, Soil nitrate nitrogen; SOM, Soil organic matter; TN, Total nitrogen; TP, Total phosphorus. A0–A9 represent *Mortierella*, unclassified fungi, *Lophotrichus*, *Melanophyllum*, *Solicoccozyma*, *Neocosmospora*, *Fungi_gen_Incertae_sedis*, unclassified *Ascomycota*, unclassified *Chaetomiaceae*, and *Aspergillus*, respectively. B0–B9 represent undefined saprotroph, endophyte–undefined saprotroph, soil saprotroph–wood saprotroph, animal pathogen, dung saprotroph–plant saprotroph, plant pathogen, plant saprotroph–wood saprotroph, fungal parasite–plant saprotroph, animal pathogen–plant pathogen–undefined saprotroph, and animal pathogen–soil saprotroph, respectively.

Redundancy analysis (RDA) was conducted to examine the relationships between soil physicochemical factors and both fungal community composition and putative fungal functional groups (Figure 9). In the RDA of soil physicochemical properties and fungal community composition, the first two ordination axes together explained 37.62% of the community variation (Figure 9a). The results indicate that TP (*r*^2^ = 0.467, *p* = 0.004), pH (*r*^2^ = 0.380, *p* = 0.018) and TN (*r*^2^ = 0.289, *p* = 0.044) are the key soil physicochemical factors influencing fungal community composition. In the RDA of soil physicochemical properties and putative fungal functional groups, the first two ordination axes together explained 40.65% of the community variation (Figure 9b). The results show that AN (*r*^2^ = 0.801, *p* = 0.001), AP (*r*^2^ = 0.544, *p* = 0.002), SOM (*r*^2^ = 0.498, *p* = 0.002), EC (*r*^2^ = 0.406, *p* = 0.012), TP (*r*^2^ = 0.325, *p* = 0.034), and NO₃^−^-N (*r*^2^ = 0.298, *p* = 0.045) are the key soil physicochemical factors affecting the distribution of putative fungal functional groups (Table 1).

## 4 Discussion

### 4.1 Effects of different treatments on soil physicochemical properties

This study demonstrates that different lavender varieties significantly alter the physicochemical properties of rhizosphere soil in semi-arid regions, indicating that varietal differences are reflected not only in aboveground growth, yield, and quality, but also in profound modifications of the rhizosphere microenvironment through root activities (Xu et al., 2026). Compared with natural soil, the rhizosphere soil pH of all three lavender varieties increased (Figure 1a), suggesting that lavender cultivation may regulate rhizosphere pH through mechanisms such as imbalanced uptake of cations and anions by roots, secretion of organic acids or alkaline substances, and alterations in microbial metabolic processes (Zheng J. et al., 2026). The electrical conductivity of rhizosphere soil significantly decreased in Xinxun No. 3 and Xinxun No. 4 (Figure 1b), indicating that these two varieties may possess stronger salt regulation or ion absorption capacities (Jia et al., 2025). In semi-arid regions, where evaporation is intense and salts tend to accumulate in surface soils, a reduction in EC helps alleviate salt stress and improve the rhizosphere environment (Lei et al., 2025). In contrast, Xinxun No. 2 showed no significant change in electrical conductivity, suggesting differences among varieties in their ability to regulate salt migration, uptake, and ionic balance in the rhizosphere (Du et al., 2026). These differences may be associated with root architecture, transpiration-driven water consumption, and the composition of root exudates (Yun et al., 2024).

Regarding nitrogen forms, all three varieties increased ammonium nitrogen content while significantly decreasing nitrate nitrogen, indicating a marked shift in nitrogen cycling within the rhizosphere (Figures 1c,d). On the one hand, plants may preferentially absorb nitrate nitrogen, leading to its depletion in the rhizosphere (Liu et al., 2025); on the other hand, microbial processes such as assimilation, denitrification, or inhibited nitrification may also contribute (Ling et al., 2022). Notably, Xinxun No. 4 significantly increased available nitrogen (Figure 1e), suggesting a more abundant pool of plant-accessible nitrogen in its rhizosphere, which may be associated with greater organic matter input and enhanced microbial mineralization activity (Ma et al., 2026). In terms of organic matter, Xinxun No. 4 showed the most significant increase (Figure 1g), suggesting a stronger capacity for root-derived carbon input, including root litter, rhizodeposits, and exudates. Increased organic matter not only improves soil aggregate structure and water- and nutrient-holding capacity, but also provides carbon sources for microorganisms, thereby further promoting nutrient transformation (Sun et al., 2025). Phosphorus dynamics exhibited more pronounced varietal effects. Although total phosphorus decreased in lavender rhizosphere soils, available phosphorus increased significantly, with the greatest increase observed in Xinxun No. 4 (Figures 1h,i). This finding suggests that lavender cultivation does not simply reduce the total phosphorus reserves in the soil; rather, it may facilitate the transformation of insoluble phosphorus into more bioavailable forms, thereby increasing the content of available phosphorus (Liu et al., 2024). Mechanisms such as root exudation of organic acids, phosphatases, and the recruitment of phosphate-solubilizing microorganisms may play important roles in enhancing phosphorus availability (Pang et al., 2024). The concurrent decrease in total phosphorus and increase in available phosphorus also reflects a dynamic balance between phosphorus activation in the rhizosphere and plant uptake. Interestingly, in the present study, when soil organic matter in the rhizosphere of Xinxun No. 4 increased significantly, total nitrogen decreased by nearly 50%. This seemingly counterintuitive pattern may be explained by the following three factors. First, Xinxun No. 4 significantly increased the contents of available nitrogen and ammonium nitrogen, indicating that although TN decreased, the bioavailable nitrogen fraction did not decline and may even have increased due to enhanced organic matter inputs and microbial mineralization. Second, the reduction in TN and NO₃^−^-N may be jointly attributed to plant nitrogen uptake, microbial assimilation and immobilization, and the rapid consumption of nitrate in the rhizosphere. Third, the increase in SOM may have been primarily derived from carbon-rich inputs such as root exudates, rhizodeposits, and fine-root residues. These carbon sources can increase soil organic matter content over a relatively short period, but may not necessarily lead to a concurrent increase in TN, particularly when they exhibit relatively high C/N ratios. Under such conditions, microbial immobilization of inorganic nitrogen may be stimulated, resulting in a temporary decoupling between SOM and TN.

Overall, different lavender varieties exhibit distinct directional and functional effects on rhizosphere soil properties. Xinxun No. 4 performed best in enhancing organic matter, available nitrogen, and available phosphorus, indicating a stronger rhizosphere fertilization effect; Xinxun No. 3 also showed advantages in reducing EC and improving certain available nutrients; whereas Xinxun No. 2 exhibited relatively weaker regulatory effects. These findings suggest that, in semi-arid lavender cultivation, varietal selection should consider not only yield and essential oil quality, but also the capacity to maintain rhizosphere soil fertility and mitigate salinity stress.

### 4.2 Effects of different treatments on soil fungal community structure and putative fungal functions

This study demonstrates that different lavender varieties can significantly reshape the structure of rhizosphere fungal communities in semi-arid regions, and that this reshaping is clearly variety-specific. This suggests that lavender does not merely passively respond to soil environmental conditions, but actively influences community assembly through root activity and rhizosphere selection processes (Park et al., 2023). Alpha diversity results showed that Xinxun No. 4 exhibited the highest richness and diversity of rhizosphere fungal communities, indicating that its rhizosphere environment is more conducive to fungal colonization and community assembly. Principal coordinate analysis (PCoA) further revealed clear separation among treatments in ordination space, suggesting that different varieties form distinct fungal communities through rhizosphere filtering effects. At the phylum level, Ascomycota dominated the community overall, reflecting the strong stress tolerance of fungal communities in arid and semi-arid environments. However, taxa such as Mortierellomycota and Basidiomycota varied significantly among varieties. LEfSe analysis showed that CK, XL2, XL3, and XL4 were enriched with different biomarker taxa, indicating that lavender varieties do not simply increase or decrease total fungal abundance, but selectively recruit or exclude specific taxa through differences in root exudate composition, nutrient (C, N, P) inputs, and rhizosphere microenvironments (Wei et al., 2026). In particular, the enrichment of genera such as Aspergillus in Xinxun No. 4, together with its higher *α*-diversity, suggests that this variety may establish a more complex rhizosphere niche (Zhang H. H. et al., 2026), thereby promoting community diversification.

FUNGuild predictions indicated that fungal functional groups across treatments were dominated by saprotrophic and endophyte–saprotroph complexes. This suggests that organic matter decomposition and nutrient recycling remain the core functions of rhizosphere fungi, serving as key biological drivers of soil fertility (Zhang G. Z. et al., 2025; Zhang S. X. et al., 2025). This finding is consistent with the ecological context of arid and semi-arid soils, where organic substrates are relatively limited and microorganisms rely more on efficient decomposition pathways (Wu et al., 2025). Compared with the natural soil control group, the proportion of Undefined Saprotrophs increased in XL2, indicating a tendency to enrich opportunistic saprotrophic fungi with strong environmental adaptability in its rhizosphere (Zheng X. N. et al., 2026). In contrast, the increased proportion of Endophyte–Undefined Saprotrophs in Xinxun No. 3 and Xinxun No. 4 suggests that varietal differences not only affect surface decomposers but may also enhance plant–fungus interactions, enabling some fungi to adopt more flexible ecological strategies between endophytic and saprotrophic lifestyles (Zheng X. N. et al., 2026). Notably, slight increases in groups such as Animal Pathogen, Plant Pathogen, and Fungal Parasite–Plant Saprotroph in Xinxun No. 4 do not necessarily indicate a higher disease risk (Peng et al., 2025), but may instead reflect a more resource-rich and complex rhizosphere niche capable of supporting a broader range of functional types (Willing et al., 2024). Notably, slight increases in groups such as Animal Pathogen, Plant Pathogen, and Fungal Parasite–Plant Saprotroph in Xinxun No. 4 do not necessarily indicate a higher disease risk, but may instead reflect a more resource-rich and complex rhizosphere niche capable of supporting a broader range of functional types. However, because FUNGuild provides putative functional assignments based on taxonomic information, these results should be interpreted cautiously. Although only ‘Probable’ and ‘Highly Probable’ assignments were retained in this study, the predicted pathogen-related guilds cannot be considered direct evidence of pathogenic activity or disease occurrence. Future studies should combine pathogen-specific molecular detection, culture-based isolation, pathogenicity assays, and metagenomic or transcriptomic validation to determine whether these predicted guilds have practical implications for lavender health.

Overall, different lavender varieties may promote a shift in fungal communities from mere compositional differentiation toward functional reassembly by altering rhizosphere resource availability and habitat heterogeneity. Among them, Xinxun No. 4 had the strongest promoting effect on community diversity and the complexity of predicted functions, highlighting its potentially greater role in rhizosphere ecological regulation.

### 4.3 Relationships among soil physicochemical properties, fungal community structure, and putative fungal functional groups

Correlation analysis and redundancy analysis (RDA) jointly indicated that the physicochemical properties of rhizosphere soil are important drivers of the restructuring of fungal community composition and changes in putative fungal functional profiles. The fungal community composition at the genus level was significantly correlated with all nine soil physicochemical indicators, indicating that nutrient availability, soil acidity–alkalinity, and salinity jointly participate in fungal community assembly and differentiation. The RDA results further showed that TP, pH and TN were the key environmental factors affecting fungal community structure, suggesting that phosphorus status and soil acidity–alkalinity play central roles in the replacement of dominant fungal taxa (Yan et al., 2026). In semi-arid soils, pH and nutrient availability are often important limiting factors for microbial growth and competition (Wang et al., 2023). Therefore, different lavender varieties may further influence fungal niche partitioning and community turnover patterns by altering rhizosphere pH and phosphorus supply (Lv et al., 2026).

At the putative functional level, AN, AP, SOM, EC, TP, and NO₃^−^-N significantly accounted for the variation in the distribution of putative fungal functional groups, with AN and AP exerting the most pronounced effects. This indicates that the supply of available nitrogen and phosphorus constitutes a key resource basis driving the change of predicted functional guilds, such as saprotrophic, endophytic–saprotrophic, and potentially pathogenic groups (Lu et al., 2025). The significant effect of SOM suggests that organic carbon input not only provides an energy source for fungal metabolism but also determines the enrichment of decomposer taxa (Zhao et al., 2024). Meanwhile, the significant influences of EC and NO₃^−^-N indicate that salinity stress and changes in inorganic nitrogen forms may alter fungal strategies for acquiring water and nutrients, thereby promoting the redistribution of functional groups among saprotrophic, symbiotic, and parasitic lifestyles (Wei et al., 2022). Combined with the FUNGuild prediction results, the treatments were generally dominated by saprotrophic and endophyte–saprotroph functional groups, whereas potentially pathogenic or parasitic taxa increased slightly in XL4. This suggests that cultivar differences may have promoted the reorganization and differentiation of fungal functions by altering rhizosphere resource conditions and microenvironmental heterogeneity (Rawat et al., 2025).

Although this study has yielded several important findings, future research should incorporate root exudate profiling, seasonal variation, and long-term field experiments to further elucidate the mechanisms through which different lavender varieties regulate rhizosphere microecology. Moreover, integrating bacterial and fungal community analyses with metagenomic sequencing and functional validation would provide a more comprehensive understanding of varietal effects on rhizosphere microbial community structure and ecological functions. The relationships between soil physicochemical properties, fungal community composition, and the distribution of predicted functional guilds should also be examined using larger sample sizes and well-controlled experiments. Collectively, these efforts would provide a more robust scientific basis for selecting superior lavender varieties and developing sustainable cultivation practices in semi-arid regions.

## 5 Conclusion

Lavender cultivar significantly influenced rhizosphere soil physicochemical properties, fungal community composition, and predicted functional guilds in the semi-arid region. Among the cultivars, Xinxun No. 4 was most effective in increasing soil organic matter, available nitrogen, and available phosphorus, while supporting greater fungal *α*-diversity. Total phosphorus, pH, and total nitrogen were the primary drivers of fungal community structure, whereas available nitrogen, available phosphorus, soil organic matter, electrical conductivity, total phosphorus, and nitrate nitrogen were closely associated with shifts in predicted fungal functional guilds. Overall, XL4 showed considerable potential to improve rhizosphere soil fertility and fungal diversity. However, its slightly higher relative abundance of predicted pathogen- and parasite-associated guilds warrants cautious interpretation and further experimental validation. These findings provide a scientific basis for lavender cultivar selection and rhizosphere soil management in semi-arid regions.

## Data Availability

The raw sequence data reported in this paper have been deposited in the Genome Sequence Archive (Genomics, Proteomics & Bioinformatics 2021) in National Genomics Data Center (Nucleic Acids Res 2022), China National Center for Bioinformation/Beijing Institute of Genomics, Chinese Academy of Sciences (GSA: CRA041854) that are publicly accessible at: https://ngdc.cncb.ac.cn/gsa.
