# Correction: Regulatory effects of lavender varieties on rhizosphere soil properties and fungal community structure and function in semi-arid regions

**DOI:** 10.3389/fmicb.2026.2014763

**Published:** 2026-10-21

**Authors:** Yongshang Tong, Yuefang Chen, Yule Li, Peiqi Duan, Shunjie Yang, Yujie Ma, Wenyan Ning, Pansheng Ge, Qianhui Li, Ruiying Qin, Na Li

**Affiliations:** 1 Xinjiang Key Laboratory of Lavender Conservation and Utilization, Yining, China; 2 College of Biological Science and Technology, Yili Normal University, Yining, China

**Keywords:** environmental driving factors, fungal community structure, lavender cultivars, rhizosphere soil microecology, semi-arid region

There was a mistake in figure as published. [The horizontal axis title of sub-graphs c and d in Figure 2 was duplicated. Moreover, the horizontal axis title of sub-graph b was written incorrectly, and the data of sub-graphs b and c overlapped. The content of sub-graph c should be Shannon index]. The corrected Figure 2 appears below.

**Figure 2:**
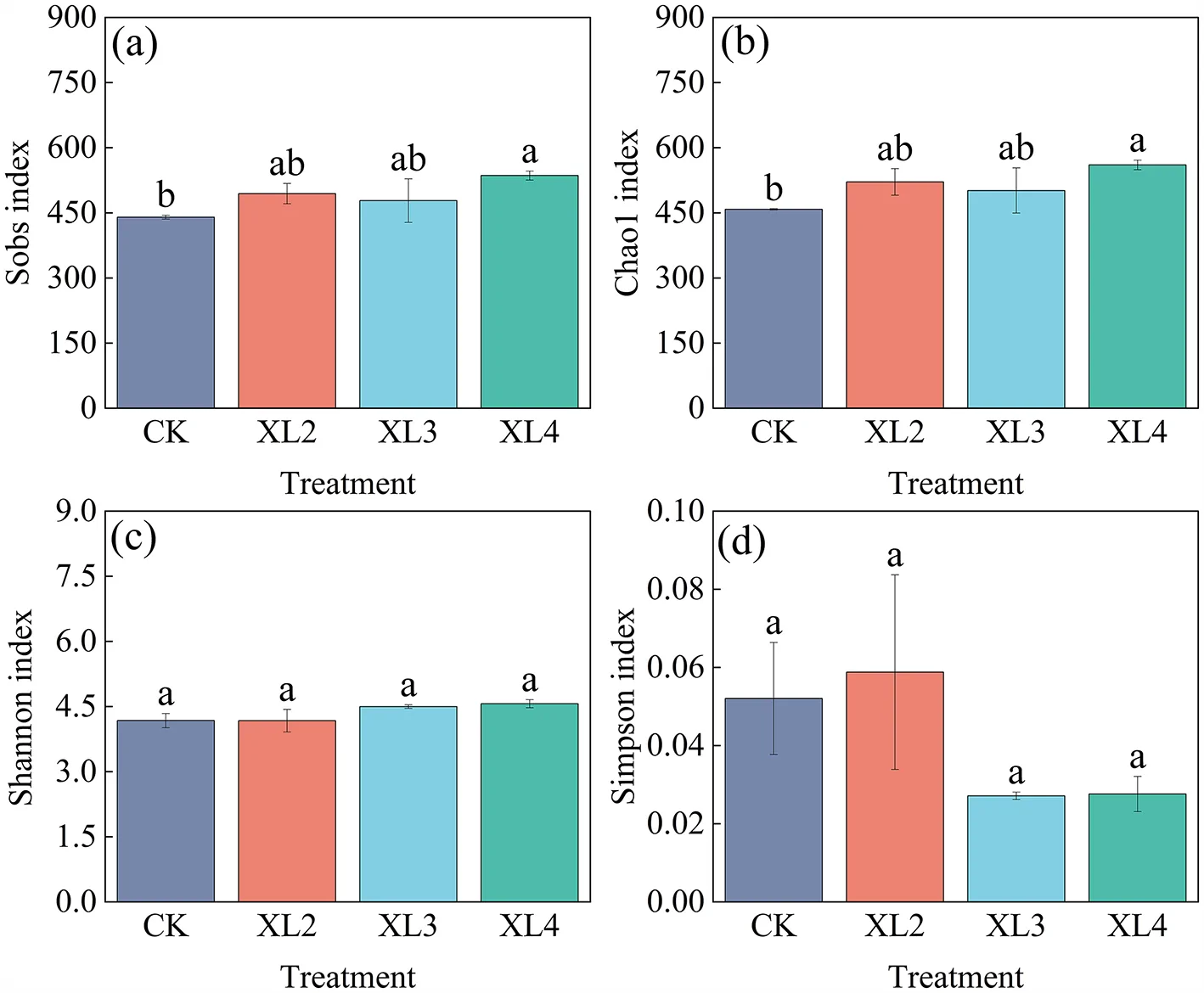
Comparison of soil fungal α-diversity. Different lowercase letters indicate significant differences among treatments. CK represents the natural soil control group; XL2 represents the rhizosphere soil of Xinxun No. 2; XL3 represents the rhizosphere soil of Xinxun No. 3; and XL4 represents the rhizosphere soil of Xinxun No. 4.

The original version of this article has been updated.

